# Dynamic deep marine oxygenation during the Early and Middle Paleozoic

**DOI:** 10.1126/sciadv.adw5878

**Published:** 2025-09-03

**Authors:** Chadlin M. Ostrander, Jean Nikolas R. Clemente, Richard G. Stockey, Justin V. Strauss, Tiffani Fraser, Sune G. Nielsen, Erik A. Sperling

**Affiliations:** ^1^ Department of Geology & Geophysics, University of Utah, Salt Lake City, UT, USA.; ^2^ Department of Geology & Geophysics, Woods Hole Oceanographic Institution, Woods Hole, MA, USA.; ^3^ School of Ocean and Earth Science, University of Southampton, Southampton, UK.; ^4^ Department of Earth Sciences, Dartmouth College, Hanover, NH, USA.; ^5^ Yukon Geological Survey, Whitehorse, Yukon, Canada.; ^6^ CRPG, CNRS, Université de Lorraine, 15 rue Notre Dame des Pauvres, 54501 Vandoeuvre lès Nancy, France.; ^7^ Department of Earth and Planetary Sciences, Stanford University, Stanford, CA, USA.

## Abstract

The Early Paleozoic radiation of diverse animal life is commonly connected to a well-ventilated global ocean. Yet the oxygenation history of Paleozoic deep oceans remains debated. Using thallium (Tl) isotope ratios in deep-marine mudrocks, we reconstruct the history of deep marine oxygenation from ~485 to 380 million years ago. Thallium isotopes can track bottom water oxygenation indirectly through their sensitivity to seafloor Mn oxide burial. We apply Tl isotopes to a global set of mudrocks, placing a particular focus on the Road River Group of Yukon, Canada. Our data reveal an oscillatory pattern in seawater Tl isotope ratios and, in turn, a dynamic ocean ventilation history. A long-lived deep ocean oxygenation episode is identified between ~405 and 386 million years ago. These short-term dynamics are superimposed on a muted positive ocean oxygenation trend over the entire Early and Middle Paleozoic. Sustained O_2_ accumulation in global marine bottom waters occurred sometime after ~380 million years ago according to our dataset.

## Teaser

Shale thallium isotope data suggest a dynamic initial ventilation of Earth’s oceans during the Early to Middle Paleozoic.

## INTRODUCTION

The initial widespread oxygenation of Earth’s deep ocean basins, referred to as ocean “ventilation,” signified a major positive increase in Earth’s surface oxygenation levels. Shallow waters in direct contact with the atmosphere oxygenate linearly with increasing atmospheric O_2_, but deeper waters not in direct contact with the atmosphere do not (*1*–*4*). The formation and subduction of cold and dense O_2_-rich waters at high latitudes, and the availability of phosphorus in global water columns, play critical roles in deep marine O_2_ accumulation (*5*). Ocean ventilation was a harbinger of a better oxygenated Earth system, perhaps signaling higher and more stable oxygen levels on the shelf for large and morphologically complex life-forms with high energetic demands.

Widespread ocean ventilation is invoked to explain nearly every important event in early animal evolution. This includes the general rise of animals during the Neoproterozoic Era [~1000 to 539 million years ago (Ma) (*6*–*8*)], the first appearance of animals in the fossil record during the Ediacaran Period [after ~580 Ma (*9*, *10*)], the Cambrian radiation [after ~539 Ma (*11*)], and the Great Ordovician Biodiversification Event [~485 to 444 Ma (*12*, *13*)]. In each instance, however, persuasive counterarguments are constructed in response (*14*–*17*). One common counterargument is that ocean oxygenation levels could have increased during some—or even all—of these important evolutionary events but perhaps not to levels high enough or for durations long enough to sustain deep ocean ventilation (*4*).

Compelling evidence for widespread ocean ventilation is emerging from the Middle Paleozoic during the Early to Middle Devonian ~420 to 380 Ma. Redox-sensitive elements and isotope ratios in Early to Middle Devonian mudrocks (*4*, *18*), carbonates (*19*–*21*), and submarine basalts (*22*) fluctuate and/or fundamentally change in a manner consistent with a better oxygenated deep ocean. These data are corroborated by the physical rock record, as the distribution of fine-grained organic-rich marine mudrocks [referred to henceforth as “shales” (*23*)] deposited beneath anoxic bottom waters on and below the continental slope becomes less abundant in the rock record around this same time (*24*). Perhaps this is when widespread and sustained ocean ventilation first occurred on Earth.

Although evidence continues to accumulate for widespread and sustained ocean ventilation much later than previously thought, the timing and tempo of this phenomenon remain largely unconstrained. Some redox records used so far to track Middle Paleozoic ocean ventilation have sparse temporal resolution [e.g., submarine basalts (*22*)]. Some of these redox records, such as redox-sensitive trace metals (*4*, *10*) and Mo isotopes (*18*), are complicated by relatively minor local-scale changes in environmental conditions that can produce false positives for oxygenation (*25*, *26*). Other redox records have a denser temporal resolution but only provide information about local- to regional-scale oxygenation dynamics [e.g., I/Ca ratios and Ce anomalies (*19*–*21*)].

To help resolve the ancient ocean ventilation debate, we reconstruct seawater thallium (Tl) isotope compositions during the Early to Middle Paleozoic from ~485 to 380 Ma. The focal point of our reconstruction is a near-continuous shale record from well-exposed outcrops of the Road River Group of Yukon, Canada. We supplement this record with a lower-resolution record from worldwide coeval shales. Our redox record is sampled at a higher resolution than most studies applied to the same time frame, uses a geochemical tool comparatively resilient to local-scale changes in environmental conditions, and has a stronger potential to specifically track widespread global ocean ventilation dynamics.

### Tl isotope paleoredox proxy

Stable Tl isotopes are well suited for tracking ancient ocean ventilation because of their unique sensitivity to Mn(IV)-bearing oxide minerals (referred to henceforth as Mn oxides) that are buried in sediments today only when O_2_ is persistently present in overlying waters (*27*). Tl has two stable isotopes, ^203^Tl and ^205^Tl, and differences in their relative abundance are reported relative to the NIST 997 Tl standard in epsilon (ε) notation as

ε205Tl(‱)=(T205/203lsample÷T205/203lNIST−997−1)×10,000

Differences in the relative abundance of stable Tl isotopes between two phases can be quantified with fractionation factors (α)

$$\alpha =R_{a}/R_{b}$$

where *R*_a_ denotes the ^205^Tl/^203^Tl ratio of the product, and *R*_b_ denotes the ^205^Tl/^203^Tl ratio of the reactant. The largest α observed to date of 1.0021, equating to an ε^205^Tl offset of +21‱ relative to seawater, is found in ferromanganese crusts and nodules and pelagic clays formed on the abyssal seafloor (*28*). The strong Tl isotope fractionation observed in these deep marine deposits is owed primarily to nuclear volume field shift effects associated with the oxidation of Tl(I) to Tl(III) after sorption to Mn oxides (*29*, *30*). Sorption experiment results suggest that the strongest Tl isotope fractionation effects are associated with the Mn oxide mineral hexagonal birnessite (*31*). Thallium sorption to other Mn oxide minerals like todorokite and triclinic birnessite is associated with a much smaller fractionation factor of 1.0001 (*32*), probably because Tl(I) is not oxidized to Tl(III) after sorption to these minerals (*30*).

The consistent presence of O_2_ in marine bottom waters is required to stabilize Mn oxide minerals in sediments and drive large net Tl isotope fractionation effects. If O_2_ is not present in bottom waters, even for as briefly as days, reductive dissolution of Mn oxides leads to the release of Mn back into the water column as Mn(II) (*27*, *33*–*35*). In sediments forming below water masses with high rates of primary productivity, for example, below coastal upwelling zones, high rates of organic matter export can even promote sedimentary Mn oxide dissolution despite the presence of O_2_ in bottom waters. Manganese oxide–driven Tl isotope fractionation effects are strongly dampened in sediments from these productive settings—sometimes, they are even erased (*36*). Even if Mn oxides are formed in the water column, no net Tl isotope fractionation can be imparted if these Mn oxides do not survive transit through the water column and long-term burial in the sediments below (*37*, *38*).

Seawater Tl isotope mass balance today is very sensitive to globally distributed Mn oxides on the seafloor. The estimated residence time of Tl in modern seawater is ~18.5 thousand years (kyr), which is longer than the ocean mixing time of ~1 kyr and results in a globally homogeneous seawater ε^205^Tl value (*39*). Today’s seawater ε^205^Tl value is −6‱ (*28*, *40*–*42*), which is lower than the global seawater input value of about −2‱ (*43*) and implies an Tl output from seawater with a strong preference for the heavier ^205^Tl isotope. This output is Mn oxide–rich sediments.

No other known primary seawater Tl outputs impart comparable isotope fractionation effects. Preferential light ^203^Tl removal during basalt alteration can drive an isotope fractionation effect up to −9‱ (*41*), although the net effect on seawater is probably much less substantial and around −1‱ (*39*). Most of the remaining seawater Tl removal today occurs in productive environments where O_2_ never penetrates sediments or only penetrates shallowly into the sediments or for short periods of time (*36*, *42*). Thallium removal in these strongly reducing settings is not associated with a quantifiable isotope fractionation effect, leading to the direct capture of the overlying seawater ε^205^Tl value. This is a finding observed in the Cariaco Basin (*42*), Black Sea (*44*), Santa Barbara Basin (*45*), Baltic Sea (*46*), and various upwelling zones (*36*). Suffice to say, water column ε^205^Tl capture occurs in a variety of sedimentary environments today and serves as a testament of Tl’s unique isotopic sensitivity to Mn oxides—and little else.

Thallium in sediments from reducing settings is commonly associated with pyrite (*47*), suggesting that pyrite and potentially also other sulfide minerals play a paramount role in sedimentary Tl retention (*37*, *42*). The kinetics of Tl removal in the presence of sulfide are extremely fast, leading to rapid Tl transfer to sediments even when sulfide is present in trace amounts (*48*). Rapid transfer is conducive to quantitative removal, a scenario that prevents the expression of any isotope fractionation effects during the removal process. This could be why sediments from reducing settings have ε^205^Tl values indistinguishable from (i.e., within the analytical error of) overlying waters [(*42*); but also see (*37*)].

The typical approach with the Tl isotope paleoredox proxy, which we also use here, is to reconstruct past seawater ε^205^Tl values from ancient fine-grained marine sedimentary rocks formed under reducing conditions. A wide range of reducing conditions has been studied with Tl isotopes, leading to a strong understanding of Tl isotope cycling within and across these environment types. This includes settings with O_2_-bearing bottom waters but with anoxic shallow sediment porewaters (*36*), settings with anoxic bottom waters and H_2_S-bearing shallow sediment porewaters (*45*), settings with ferruginous bottom waters and sediment porewaters (*38*), and euxinic settings with abundant H_2_S in bottom waters and sediment porewaters (*37*, *42*, *44*, *46*). Sediments formed in each of these reducing settings are shown to be capable seawater ε^205^Tl archives. Changes to past seawater ε^205^Tl values are driven most efficiently by changes in the global extent of sedimentary Mn oxide burial (*42*, *49*). Isotope compositions that are unfractionated relative to global inputs (ε^205^Tl ≈ −2) would indicate little to no sedimentary Mn oxide burial on a largely anoxic global seafloor [e.g., during most of the Precambrian (*50*–*54*)]. Vice versa, lower ε^205^Tl values would support expanded seafloor Mn oxide burial [compare today and during the geologically recent past (*55*)].

### Sample background

**Fig. 1.**
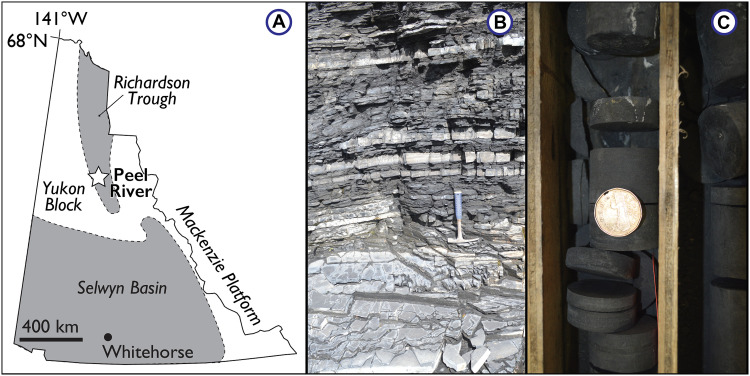
Sample context for Road River and Earn group shales. (**A**) Simplified paleogeography of the area surrounding the Peel River locality. (**B**) Basinal shales and limestones of the upper Silurian (Pridoli) Vittrekwa Formation, Road River Group, exposed on the Peel River, Yukon. (**C**) Basinal cherts and siliceous shale of the Middle Devonian Canol Formation, Earn Group, sampled from the RI-07-07A core, Yukon. Loonie for scale. Photo credit to E.A.S. for (B) and (C).

We focus our study on shales collected from the Early to Middle Paleozoic Road River and Earn groups of Yukon, Canada (*n* = 82) (Fig. 1). These strata and the associated samples are described in detail in previous work (*56*, *57*). Briefly, samples were collected from the deep-water Peel River locality within the Richardson Trough, with samples of the Cambrian-Devonian Road River Group collected from outcrop and samples of the Devonian Earn Group (Canol Formation) collected from the RI-07-07A core. Outcrops from the Peel River locality were freshly exposed during Late Quaternary to Holocene fluvial incision and remain largely unoxidized because of their preservation within the subarctic. The Road River Group is dominated by organic-rich fine-grained carbonate (sometimes secondarily silicified) and siliciclastic strata deposited in slope and basin-floor environments (*56*, *58*, *59*). It is separated from the overlying Canol Formation of the Earn Group by a highly condensed interval and several “Hyper-Enriched Black Shale” (HEBS) horizons that contain economic-grade Ni-Mo-Zn-Pt-Pb-Au-Re mineralization (*60*, *61*). The most prominent HEBS horizon, which is dated to the Eifelian-Givetian boundary and can be found regionally across Yukon, occurs at the Road River Group-Canol Formation contact and yields geochemical and paleontological evidence for a seawater (scavenging) origin for the metal enrichments (*61*, *62*). The Earn Group likely records a change in basin type, specifically deposition in a distal foreland basin related to the onset of the Ellesmerian orogeny in Arctic Canada. The laminated to massive cherts and shales of the Canol Formation also record deep-water sedimentation.

**Fig. 2.**
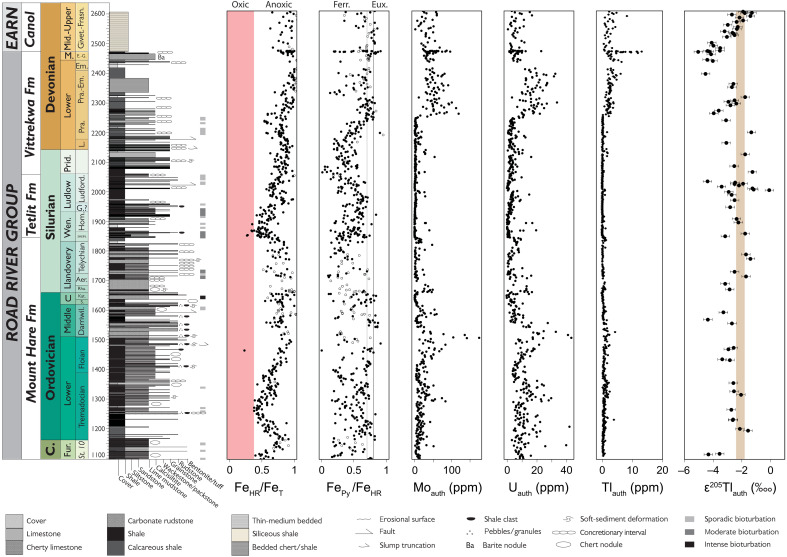
Geochemical data for Yukon shales from the Road River and Earn groups. Stratigraphic heights refer to the full composite section on the Peel River described by Strauss *et al.* (*56*) and continue into the Canol Formation in the RI-07-07A core. From left to right, the first two columns show the results of highly reactive iron–to–total iron (Fe_HR_/Fe_T_) and pyrite–to–highly reactive iron (Fe_P_/Fe_HR_) speciation analyses, interpreted using accepted empirical baselines for oxic, anoxic, euxinic, and ferruginous conditions (*63*). The few samples with Fe_T_ < 0.5 wt % or Fe_HR_/Fe_T_ > 1 are denoted as open symbols. This is followed by authigenic Mo, U, and Tl concentration data (*57*). The final column is authigenic Tl isotope data. Errors bars associated with the Tl isotope data signify the 2SD reproducibility of repeat sample measurements or the USGS SCo-1 standard, whichever is greater.

**Fig. 3.**
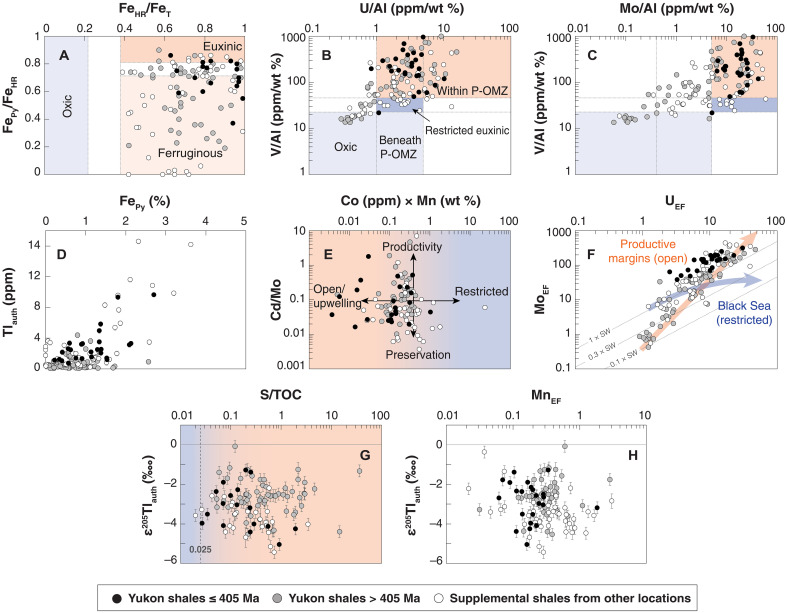
Geochemical data from shales targeted in this study that provide additional context about the ancient depositional environment. Iron speciation and redox-sensitive elements commonly used to track local redox conditions suggest predominantly anoxic local redox conditions at the time of formation (**A** to **C**). Sedimentary pyrite is a prominent Tl host phase for the shales (**D**), whereas Mn oxide minerals are/were not (**H**). The data also suggest that the ancient depositional site was not a strongly restricted basin (**E** and **F**; also see B and C), and that high organic carbon contents are unlikely to be responsible for low e205Tl values (**G**). In general, samples with data that plot within the red fields are more ideal for Tl isotope paleoredox proxy applications than those that plot within the purple fields. See Discussion for more detailed discussion. Our data is plotted in a manner similar to how the data is plotted in Bennett and Canfield (*65*) (P-OMZ, perennial oxygen minimum zone), from Sweere *et al.* (*76*), and from Algeo and Tribovillard (*64*).

Previous geochemical studies, combined with field observations that bioturbation and in situ benthic fauna are rare to absent, have demonstrated that shales from the Road River and Earn groups were deposited under anoxic conditions ideal for seawater ε^205^Tl capture (*56*, *57*). A large fraction of the total Fe (Fe_T_) in all shale samples is hosted in highly reactive phases (Fe_HR_) (Figs. 2A and 3A). All shale samples targeted in this study have Fe_HR_/Fe_T_ ratios that exceed 0.38, a threshold shown in modern and recent sediments to indicate anoxic bottom waters (*63*). A moderate fraction of this Fe_HR_ pool is composed of pyrite-hosted Fe (Fe_Py_) (Figs. 2B and 3A). Some shales have high Fe_Py_/Fe_HR_ ratios that indicate deposition under locally anoxic and sulfide-bearing “euxinic” bottom waters (Fe_Py_/Fe_HR_ > 0.70 or 0.80), but most have lower ratios indicative of locally anoxic and Fe(II)-bearing, or ferruginous, conditions (*63*). Moderate Mo and U enrichments are also consistent with locally reducing conditions (Fig. 2, C and D) because both elements are efficiently sequestered in sediments that form under these conditions (*64*). Aluminum-normalized shale V, Mo, and U concentrations are comparable to those found in sediments today formed within oxygen minimum zones (Fig. 3, B and C) (*65*). Total organic carbon (TOC) contents are elevated throughout the studied interval and also support locally reducing conditions, averaging 2.6 wt % and reaching as high as 8.8 wt % (*57*).

The remainder of our Early Paleozoic shale samples (*n* = 44) come from other geographic locations. These supplemental samples come from the Aizpute-41 core in Latvia; the E1-NC174 core in Libya; the Welsh Basin in the United Kingdom; the Franklinian passive margin in Nunavut, Canada; the Kotaneelee I-48 core from the Liard basin of Yukon; the WV-7 core from the Appalachian Basin in West Virginia (US); and the Sappington Basin in Montana (US). Details on the geologic setting of these samples are published elsewhere (*57*, *66*–*71*). These shales also have Fe_HR_/Fe_T_ ratios indicative of locally anoxic sedimentation (Fig. 3A), an inference once again supported by elevated TOC (averaging 4.6 wt %) and redox-sensitive trace metals (Fig. 3, B and C).

## RESULTS

**Fig. 4.**
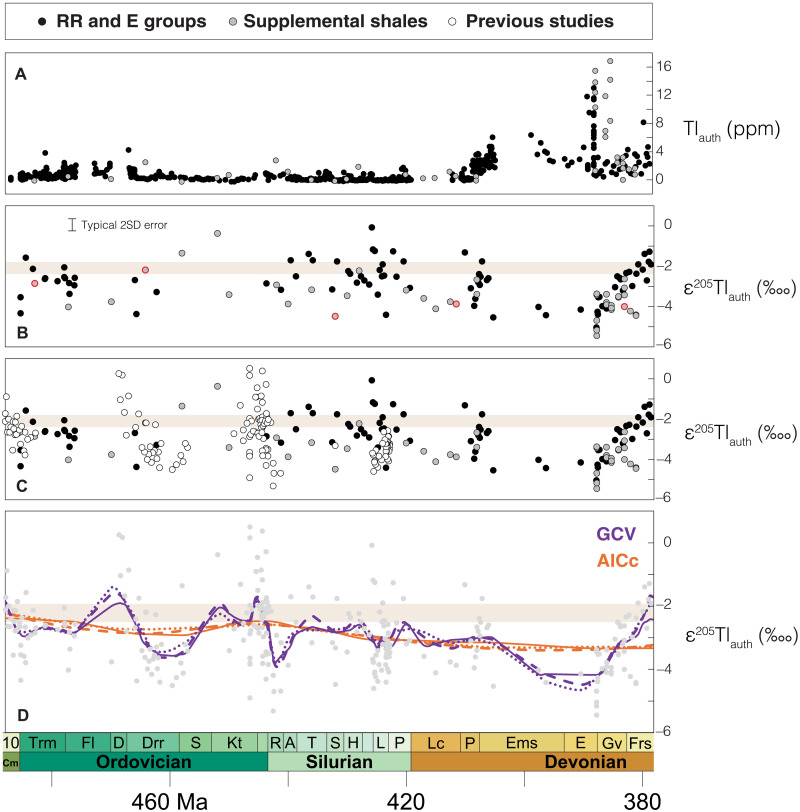
Compiled and modeled Early to Middle Paleozoic shale Tl data. (**A**) Authigenic Tl data calculated for shales targeted in this study. (**B**) ε^205^Tl_auth_ for shales targeted in this study. Data points with a red border do not pass the Mn-Ba-U decision tree established by Wang *et al.* (*36*). The typical 2SD error on Tl isotope ratio data is 0.3‱. (**C**) ε^205^Tl_auth_ for shales targeted in this study and for shales targeted in previous studies from the same time frame (*13*, *73*–*75*). (**D**) LOESS regression model results. Line styles indicate the number of polynomials: solid, 0; fine dash, 1; coarse dash, 2. RR, Road River. E, Earn.

Tl concentration data for all Peel River shales were previously reported (*57*). To maintain consistency with previous work on the same samples, all bulk-rock Tl concentrations are converted to authigenic seawater-derived concentrations (Tl_auth_) using Tl/Al ratios and compositional estimates for the bulk upper continental crust [Tl/Al_crust_ = 1.1 × 10^−5^; (*72*)]. Authigenic Tl concentrations exhibit a stepwise increase from 0.4 parts per million (ppm; *n* = 570) to 3.2 ppm (*n* = 136) in Pragian (Lower Devonian, ~411 to 408 Ma) shales of the Road River Group (Fig. 2E). Especially high Tl_auth_ values are found in the several meters of strata beneath the Eifelian-Givetian HEBS layer, reaching up to 13.0 ppm in samples with estimated ages of ~389 to 388 Ma. None of the samples collected near the HEBS layer exhibited any signs of synsedimentary mineralization. Comparable Mo_auth_ and U_auth_ trends are found in the same samples (Fig. 2, C and D) (*57*). Similar Tl_auth_ trends are also found in our supplemental samples from other locations (Fig. 4A). This includes especially high Tl_auth_ values in the Givetian Marcellus Shale, reaching up to 16.6 ppm in samples with estimated ages of ~388 to 385 Ma (Fig. 4A).

All Tl isotopic data represent authigenic values leached from sedimentary sulfides (see Materials and Methods), minerals shown in reducing settings to have ε^205^Tl values indistinguishable from overlying waters (*36*, *37*, *42*, *44*–*47*). Leached Tl_auth_ values are weakly correlated with Fe_Py_, in broad support of a primary sulfide host for Tl_auth_ across the stratigraphically extensive and geographically diverse sample set (Fig. 3D). Authigenic ε^205^Tl (ε^205^Tl_auth_) values from the lower Road River Group are variable over short stratigraphic intervals but average −2.6 ± 1.7‱ (2SD) and are thus, on the whole, generally comparable to the average composition of the bulk upper continental crust (Fig. 2F). In contrast, Lower and Middle Devonian samples from ~405 to 386 Ma reveal consistently lower ε^205^Tl_auth_ values that average −4.2 ± 0.9‱ (2SD; *n* = 11). Above this in the Canol Formation, ε^205^Tl_auth_ values increase progressively, returning to crustal values in samples with estimated ages of ~378 Ma and younger to the top of the measured section. Tl isotope data from the supplemental samples are comparatively complex but do reveal some consistencies with the Road River Group shales (Fig. 4B). Shales deposited before ~405 Ma have average ε^205^Tl_auth_ values of −3.1 ± 2.0‱ (2SD), whereas shales deposited ~405 to ~386 Ma have slightly lower averages of −4.0 ± 1.3‱ (2SD). The lowest of these ε^205^Tl_auth_ values, down to −5.4 ± 0.3‱, is found in a sample estimated at ~388 Ma from the Marcellus Shale, a unit that reveals strong Tl isotope (and Tl_auth_) consistencies with the time-equivalent (Givetian) Canol Formation samples (Fig. 4, A and B).

## DISCUSSION

### Compiled Early and Middle Paleozoic shale ε^205^Tl records

To gain a more comprehensive picture of deep ocean oxygenation during the Early and Middle Paleozoic, we also take into consideration other published shale Tl isotope datasets from across the same time frame (Fig. 4C) (*13*, *73*–*75*). We assume, in accordance with how these data were originally interpreted, that these studied successions are also viable seawater ε^205^Tl archives.

To form continuous quantitative estimates of seawater ε^205^Tl values during the Early and Middle Paleozoic, we apply cross-validated locally estimated scatterplot smoothing (LOESS) regression models to the compiled data (Fig. 4D). Two cross-validation approaches were applied: generalized cross-validation (GCV) and Akaike information criterion (AICc, where the lowercase “c” signifies a correction for small sample sizes). The GCV models capture averaged short-term changes in seawater ε^205^Tl values over a single million to a few tens of millions of years. By contrast, the AICc cross-validated LOESS models track longer term changes in seawater ε^205^Tl values over many tens of millions of years.

According to the GCV regression model, seawater ε^205^Tl was highly variable across the studied time frame, fluctuating on the order of ~1 to ~2‱. The average temporal resolution of the ε^205^Tl_auth_ data is one sample every ~450 kyr, which is longer than Tl’s seawater residence time today of ~18.5 kyr (*39*). Hence, seawater ε^205^Tl could have been even more variable than the compiled data suggest [compare (*36*)]. The protracted negative ε^205^Tl excursion down to −5.4‱ between ~405 and 386 Ma is a prominent feature in the GCV model and could record an especially strong and long-lived ocean oxygenation episode. The estimated time frame of this episode broadly overlaps with other independent lines of geochemical evidence for enhanced ocean oxygenation during the Middle Devonian (*4*, *18*, *22*, *24*). The return to near-crustal ε^205^Tl values after this excursion is also intriguing and could mark the return to a more anoxic global deep ocean between ~386 and 378 Ma. This age broadly overlaps in time with a decline in worldwide carbonate I/Ca ratios, which was also interpreted to mark expanded marine anoxia (*20*).

According to the AICc regression model, seawater ε^205^Tl decreased slightly with time, from about −2‱ in the Latest Cambrian to about −3‱ in the Middle Devonian. This trend could track a muted and much broader net oxygenation of Earth’s oceans over the studied time interval.

### Seawater ε^205^Tl archive fidelity

Restricted basins are the Achilles heel of the Tl isotope paleoredox proxy. Sediments formed under anoxic conditions in these environments today still capture overlying water column ε^205^Tl values, but these seawater values are skewed to higher ε^205^Tl values that better match localized inputs [e.g., local rivers and anthropogenic sources (*37*, *42*, *46*)]. While it is possible that the Road River Group samples were affected by basinal restriction, this intraplatformal trough is thought to have been open to the north and south (*58*) and more comparable hydrologically to the modern Exuma Sound or Tongue of the Ocean in the Bahamas (*56*). Aluminum-normalized shale V, Mo, and U concentrations better match those found in sediments today formed within oxygen minimum zones than those formed in highly restricted basins (Fig. 3, B and C) (*65*). Note that these comparisons (and others that follow) do not take into consideration past differences in the size of global seawater reservoirs for these elements. Combined abundances of Co and Mn in the shales are generally low and seemingly are also better aligned with sediments formed in nonrestricted settings (Fig. 3E) (*76*), as are quasilinear and strongly sloped Mo and U enrichment factor patterns (Fig. 3F) (*64*). The 11 Road River shale samples formed between ~405 and 386 Ma with lower ε^205^Tl_auth_ (black data points in Fig. 3) do not have geochemical patterns that deviate from those found in the remainder of the Road River Group shales (gray data points in Fig. 3), serving as evidence against local controls being the primary driver of the observed negative ε^205^Tl_auth_ excursion. The Marcellus Shale, which was deposited in a geographically distinct basin, shows the same negative ε^205^Tl_auth_ excursion, among other geochemical trends.

It is unlikely that the Road River Group shales were affected by regional HEBS mineralization. Thallium isotope data collected from a nearby location in Yukon across the HEBS horizon are generally consistent with our dataset (*77*). In Crawford *et al.*’s work (*77*), low ε^205^Tl values between about −4.0 and −7.0‱ were found in upper Road River Group shales, whereas higher ε^205^Tl values between about −4.5 and −2.0‱ were found in the overlying Canol Formation. In the HEBS horizon itself, Crawford *et al.* (*77*) found ε^205^Tl values below −6.0‱ and interpreted these values as being seawater derived. More work is warranted on the origin of these HEBS deposits and their potential relationship to Tl sequestration, but at present, ε^205^Tl values recovered from our samples, and even the HEBS horizon itself, seem largely unaffected by synmineralization or postdepositional processes.

Preferential ^203^Tl delivery to local sediments, manifested as low ε^205^Tl_auth_, was recently observed in a modern ferruginous lake [Deming Lake in MN, US (*38*)]. Many samples from the Road River and Earn groups were formed under locally ferruginous conditions according to Fe speciation and Mo and U trace metal data (*57*). In sediments from the modern ferruginous lake, ^203^Tl delivery by some combination of biomass and organic S is hypothesized to drive the lower sediment ε^205^Tl_auth_ values. The low ε^205^Tl_auth_ values appear most prominently in lake sediments with very low S/TOC ratios (<~0.025), presumably because efficient sequestration of unfractionated Tl in sediments with trace amounts of dissolved sulfide masks the lower ε^205^Tl_auth_ signal from organics. However, our Paleozoic shale samples have comparatively elevated S/TOC ratios (Fig. 3G), making them unlikely carriers of low ε^205^Tl_auth_ signals from the same processes observed in the modern ferruginous lake. The results of Ostrander *et al.* (*38*) suggest that Fe cycling in ferruginous Deming Lake does not impart any obvious direct effect on Tl isotopes. For this reason, and consistent with other reducing settings studied to this point (*36*, *37*, *42*, *44*–*46*), seawater ε^205^Tl capture seems likely for our shales formed under ferruginous conditions.

Last, seawater ε^205^Tl capture is supported throughout our sample set by a recently calibrated decision tree test that uses crust-normalized trace metal enrichments of Mn, Ba, and U to identify sediments that capture seawater ε^205^Tl values (*36*). The decision tree is formulated on core-top sediment data from productive upwelling zones with variable bottom water O_2_ contents and sediment porewater geochemistry. It identifies local redox conditions more conducive to sulfide formation than Mn oxide burial. The former leads to seawater ε^205^Tl capture, whereas the latter leads to ^205^Tl enrichment. When the decision tree is applied to our shales, positive results are recovered for 121 of 126 samples (96%). None of the shales that give negative results are from the Road River and Earn groups; negative results are only recovered from the supplemental shale dataset. None of these few negative results are concentrated at a particular site or time frame (illustrated in Fig. 4), and their omission does not change our preferred interpretations. No obvious correlations are found between ε^205^Tl_auth_ values and Mn enrichment factors (Fig. 3H), a finding also inconsistent with ^205^Tl enrichment via local Mn oxide burial.

If the seawater Tl residence time was shorter than the ocean mixing time during the Early Paleozoic, then seawater ε^205^Tl values captured by shales would potentially not be globally representative but instead regionally or locally representative [compare the modern Black and Baltic seas (*42*, *46*)]. This scenario would severely limit our ability to infer global ocean oxygenation dynamics. According to results from a Tl isotope mass-balance model, the seawater Tl residence time would only have been shorter than the ocean mixing time if an unrealistic area of the global seafloor was overlain by euxinic waters [>20% (*53*)]. Also note the broad, and at times even very strong (across the Eifelian-Givetian boundary), ε^205^Tl agreement found in our worldwide shales (Fig. 4B). A short seawater Tl residence time cannot be entirely ruled out but does not seem strongly supported by mass-balance models or the available Tl isotope data.

### Tl concentration trends

Higher Tl_auth_ values in the Road River Group shales younger than ~410 Ma likely track a combination of local and global factors. At a local scale, these data could indicate the development of more reducing redox conditions within the Richardson Trough. Higher Fe_Py_/Fe_HR_ ratios are also found around this same stratigraphic level (Fig. 2B), indicating a larger fraction of Fe hosted in sulfide minerals amenable to sedimentary Tl sequestration. High Tl_auth_ values are also found in broadly coeval samples from the Marcellus Shale of the Appalachian Basin (Fig. 4A). At a global scale, this coeval Tl_auth_ increase could track the development of a larger dissolved seawater Tl reservoir. This scenario would require some combination of increased global seawater Tl delivery rates and/or decreased global seawater Tl removal rates, both of which are challenging to differentiate with the available data. Note that a contraction of euxinic conditions, for example, in parallel with the widespread oxygenation potentially identified at this time, would be conducive to a larger dissolved seawater Tl reservoir.

### Case for a deep ocean oxygenation record

**Fig. 5.**
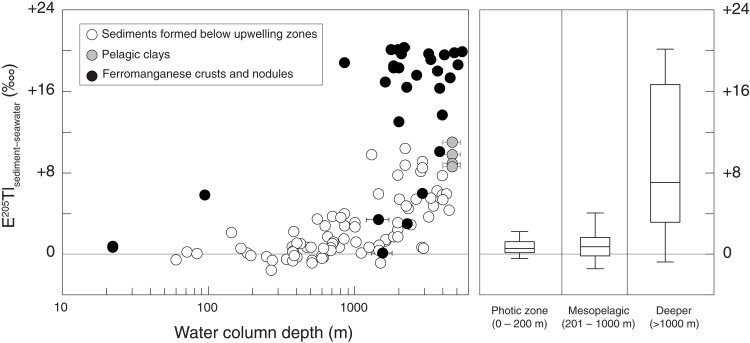
Biplot showing the depth dependence of Tl isotope fractionation in modern sediments. The *y* axis represents the ε^205^Tl difference between the sediment and seawater, and the *x* axis represents the water column depth above the sediment. White data points signify samples collected below various productive upwelling zones (*36*). Gray data points signify pelagic clays (*96*). Black data points signify ferromanganese nodules and crusts (*28*, *96*). Box and whisker plots are provided to summarize data from sediments formed in the photic zone (0 to 200 m), in the mesopelagic zone (201 to 1000 m), and on the deeper seafloor (>1000 m).

Does our Tl isotope dataset track changes specifically to deep ocean oxygenation levels? Short-term changes in oxygenation levels exclusively in the shallow photic zone (0 to ~200 m) are unlikely to explain the ε^205^Tl variability because sediments formed at these depths today do not cover a large area of the seafloor and they do not typically impart large magnitudes of Tl isotope fractionation. Data are limited (*n* = 10), but sediments from these sites have an average ε^205^Tl offset from seawater of +0.98‱ (Fig. 5) (*36*). The dissolved O_2_ content of these shallowest waters scales linearly with atmospheric O_2_, and atmospheric O_2_ does not seem to have been comparatively variable over the same timescales during the Early to Middle Paleozoic (*78*–*80*). Although note that the longer termed ε^205^Tl excursion between ~405 and 386 Ma could potentially be made temporally consistent with some inferences of Middle Paleozoic atmospheric O_2_ variability (*80*, *81*).

Fluctuating O_2_ levels in the mesopelagic zone (~200 to 1000 m) are more plausible but still unlikely to explain the data. Dissolved O_2_ contents in these mid-depth waters are more strongly controlled by organic carbon export rates, which, when high, can drive swift and substantial water column O_2_ depletion without affecting atmospheric O_2_ (*82*). However, and like in the photic zone, sediments formed under mesopelagic waters constitute only a very small area of seafloor and oftentimes do not strongly fractionate Tl isotopes (Fig. 5). Tl isotope fractionation in these settings is limited because organic carbon export promotes Mn oxide dissolution at or shortly below the sediment-water interface (*36*). Sediments formed within the mesopelagic zone targeted for Tl isotopes by Wang *et al.* (*36*) impart an average ε^205^Tl offset of +0.8. Given their small areal extent and limited magnitude of isotope fractionation, sediments formed beneath mesopelagic and photic zone waters are unlikely to explain the ~1 to ~2‱ variability observed in the Early Paleozoic shale data. Low ε^205^Tl values down to −5.4‱ at ~388 Ma are especially difficult to explain with only sediments from these areas.

Tl isotope fractionation effects imparted by seafloor sediments formed below 1000 m are more than sufficient to explain the range of ε^205^Tl values found in the Paleozoic shale data. Sediments formed beneath productive waters below the 1000-m water depth impart an average ε^205^Tl offset of +4.0‱, a factor of 5 larger than sediments formed in the mesopelagic zone (Fig. 5). Ferromanganese crusts and nodules generally form at even greater depths [commonly 4000 to 6500 m deep in the modern ocean (*83*)] and impart even greater Tl isotope fractionation effects up to +21‱ (visible in Fig. 5). Sediments formed at these greater depths are more likely to bury Mn oxides over long timescales today because bottom water dissolved O_2_ levels are more stable, and organic carbon export and sedimentation rates are typically lower. All three factors are conducive to the stabilization of Mn oxides in sediments—and hence also the generation of large Tl isotope fractionation effects.

Deep O_2_-rich waters form at cold high latitudes where gases are more soluble and relaxed density gradients permit the subduction of these O_2_-rich waters into the deep ocean. And once dissolved O_2_ makes it to the organic carbon–poor deep ocean, it is less likely to be consumed during organic matter remineralization. With this in mind, any processes that (i) decrease the dissolved O_2_ contents of high-latitude waters (e.g., higher polar seawater temperatures or lower O_2_ partial pressures in the atmosphere), (ii) decrease the efficiency of ocean circulation (e.g., weaker density gradients at the poles or changing paleocontinental configurations), or (iii) increase the efficiency of O_2_ consumption in deep waters (e.g., higher organic carbon export rates) would have had a negative impact on deep ocean O_2_ levels. These processes need not be mutually exclusive, and some combination of them operating over timescales of hundred thousands to millions of years could have driven the deep ocean oxygenation dynamics inferred from our dataset. Changes in continental configurations, for example, are shown capable in recently constructed models of driving changes to these processes that would promote temporally dynamic ocean ventilation during the Paleozoic even under modern atmospheric O_2_ levels (*84*).

According to a Tl isotope mass-balance model constructed from existing constraints and applied in an earlier study, large negative shifts in seawater ε^205^Tl values are only possible when the global area of oxygenated seafloor exceeds ~30% (*53*). Qualitatively, it is notable that our lowest seawater ε^205^Tl estimate, down to −5.4‱ at ~388 Ma, is comparable to seawater ε^205^Tl values reconstructed during the Last Glacial Maximum when ocean ventilation was only slightly weaker than today (*55*). According to the Li *et al.* (*53*) mass-balance model, the especially low ~388 Ma seawater ε^205^Tl value equates to an area of oxygenated seafloor much higher than 30%, extending past continental shelves and slopes and onto the deep seafloor where strong Tl isotope fractionation effects are more common (Fig. 5).

### Dynamic Paleozoic ocean ventilation

Multiple independent lines of geochemical evidence support a better oxygenated deep ocean at around 400 Ma (*4*, *18*–*22*, *85*). Our Tl isotope data also seem to support this, providing evidence for an especially strong episode of ocean oxygenation at around 405 to 386 Ma. However, this was no simple and binary change; our Tl isotope data also provide evidence for widespread ocean deoxygenation immediately after at around 386 to 378 Ma. Other lines of geochemical evidence support this finding, too [e.g., I/Ca ratios and Ce anomalies in carbonates (*20*, *21*)].

Evidence of widespread ocean anoxia is also found in younger sedimentary rocks formed during the Later Devonian and Early Carboniferous (*86*). At least some of these anoxic events were widespread, such as that hypothesized at the Frasnian-Fammenian boundary (*87*, *88*). However, others may have been more diachronous and regional (*71*, *89*). Some of the higher ε^205^Tl_auth_ values in our shales, for instance, from the Fammenian Sappington Formation, could represent a combination of widespread regional anoxia and/or Later Devonian anoxic events.

All data considered, the ventilation history of Earth’s oceans was more complex than classically thought (*8*). Classically, it was thought that full oxygenation of the Earth system occurred in the Late Neoproterozoic, while more recently, it has been recognized that this likely occurred closer to the Devonian (*4*, *18*, *90*). It is important to not replace a simple conceptual Neoproterozoic step-change oxygenation model with a simple Devonian step-change oxygenation. The data reported here suggest that Paleozoic oxygenation was not a binary and/or rapid phenomenon but instead one that unfolded in fits and starts over many tens or hundreds of millions of years. This seems to be an emerging theme of Earth’s oxygenation story; the initial rise of O_2_ across the Archean-Proterozoic boundary likely also unfolded in fits and starts over several hundreds of millions of years (*91*–*93*).

When did O_2_ first accumulate in the deepest portions of Earth’s oceans for extensive periods of geological time? According to new and compiled shale Tl isotope data, this happened only transiently during the Paleozoic between ~485 and 380 Ma. We do not find evidence of a sharp or sustained O_2_ accumulation in Earth’s deep oceans across this timeframe. Instead, we find evidence of dynamic ocean ventilation over a single million to a few tens of millions of years. These short-term dynamics may have been superimposed on a broader trend of deep ocean oxygenation over many tens of millions of years.

Our data allow us to identify a potential, especially strong deep ocean oxygenation episode between about 405 and 386 Ma that was more substantial than any point in the Ordovician or Silurian. However, even this “episode” seems punctuated by widespread ocean anoxia. More evidence of the same is emerging from geochemical trends recovered from slightly younger Devonian sedimentary rocks in other studies. The timing of fully sustained deep ocean oxygenation on Earth remains unknown but occurred after ~380 Ma according to our Tl isotope data. Shales immediately preceding Mesozoic oceanic anoxic events reveal lower ε^205^Tl_auth_ values consistent with a fully and stably oxygenated deep ocean [ε^205^Tl_auth_ ≈ −5 to −6‱ (*94*, *95*)]. However, the focused timescales of these event-specific studies mean that they represent fleeting snapshots in geological time. Long-term and densely sampled global redox records, such as presented here for the Early and Middle Paleozoic, are required from the Late Paleozoic and Mesozoic to fully elucidate the ventilation history of Earth’s deep ocean.

## MATERIALS AND METHODS

All shale samples were subjected to gentle leaching shown in previous work to effectively isolate the authigenic Tl hosted primarily in pyrite from detrital Tl (*47*). About 80 mg of powdered shale from each sample was weighed into an acid-cleaned perfluoroalkoxy alkane (PFA) vial, leached overnight in 2 M HNO_3_, and thereafter centrifuged. After centrifugation, supernatants were transferred to new acid-cleaned PFA vials and dried to completion on a hotplate. Once completely dry, the samples were amended with a 5-ml solution of inverse aqua regia and placed on a hotplate overnight at 135°C. Samples were then dried to completion, amended in a 6-ml solution of concentrated HNO_3_ and H_2_O_2_ (at a ratio of 5:1) to help break down organics, and placed on a hotplate overnight at 135°C. This acid attack was repeated until samples no longer stuck to the interior of the PFA vials. Once the samples were free of obvious organics, they were amended in a 5-ml solution of 1 M hydrochloric acid and heated overnight at 100°C in preparation for ion exchange chromatography.

All samples were purified from matrix elements using a previously established two-column technique (*39*). The day before the first column, each sample was amended with brominated water to oxidize all of Tl(I) in the sample to Tl(III). Samples were passed through a large glass column packed with 1.5 ml of AG1-X8 anion exchange resin. The samples were then dried to completion and amended with 1 ml of concentrated HNO_3_ and H_2_O_2_ (at a ratio of 9:1) to break down organics eluted from the column. Samples were then amended with 1 ml of 1 M HCl and heated for ~5 hours on a hotplate at 100°C in preparation for the final column. Each sample was again amended with brominated water the day prior. The second column used a much smaller Teflon microcolumn packed with 0.1 ml of AG1-X8 resin. After the column, each sample was again dried and amended with 1 ml of concentrated HNO_3_ and H_2_O_2_ (at a ratio of 9:1) to break down organics. After this step, samples were dried to completion and amended with 0.5 ml of 0.1 M HNO_3_ and 0.1% sulfuric acid in preparation for isotope ratio measurement.

Thallium isotope ratio measurements were performed using a Thermo Finnigan Neptune multicollector inductively coupled plasma mass spectrometer located at the WHOI Plasma Facility. An Aridus II desolvating nebulizer system was used during sample introduction. Measurements were performed in low-resolution mode using sample-standard bracketing and external normalization to NIST SRM 981 Pb. The Tl isotope compositions are reported relative to NIST SRM 997.

The amount of Tl present in each sample was ≥10 ng (oftentimes much greater), which was much higher than 0.0002 ng of the procedural blank. All samples were analyzed in duplicate at a concentration of ~7 ng/g Tl, which typically yielded an ion current around 70 pA on a mass/charge ratio of 205 (10^11^-ohm resistor). The average and maximum 2SD reproducibility values of these duplicate measurements were 0.1 and 0.5‱, respectively. One USGS shale SCo-1 standard was leached, purified, and analyzed with each sample set to monitor accuracy. This standard yielded an ε^205^Tl_auth_ value of −3.0 ± 0.3‱ (2SD; *n* = 5), which is indistinguishable from values reported in previous work for the same standard material [ε^205^Tl = −3.0 ± 0.2‱ (2SD) (*50*)]. Reported errors for our samples measured in duplicate are always in 2SD and either equal to the reproducibility of SCo-1 or the individual sample’s reproducibility, whichever is greater.
