# Supplementary material for: Dynamic deep marine oxygenation during the Early and Middle Paleozoic
Source: Sci Adv. 2025 Sep 3;11(36):eadw5878. doi: 10.1126/sciadv.adw5878 (PMC12407078; doi:10.1126/sciadv.adw5878)
Supplement: Supplementary file 1 — Legend for table S1 [file sciadv.adw5878_sm.pdf]

Science Advances
AAAS

Supplementary Materials for

Dynamic deep marine oxygenation during the Early and Middle Paleozoic

Chadlin M. Ostrander *et al.*

Corresponding author: Chadlin M. Ostrander, chadlin.ostrander@utah.edu

Sci. Adv. **11**, eadw5878 (2025)
DOI: 10.1126/sciadv.adw5878

The PDF file includes:

Legend for table S1

Other Supplementary Material for this manuscript includes the following:

Table S1

Table S1.
Full geochemical dataset with new and previously published data. This file includes all new and literature-compiled geochemical data.
